# Premature Infant Gut Microbiome relationships with childhood behavioral scales: preliminary insights

**DOI:** 10.3389/fnut.2023.1294549

**Published:** 2024-02-14

**Authors:** Samia Valeria Ozorio Dutra, Anujit Sarkar, Ji Youn Yoo, Emily Shaffer-Hudkins, Maureen Groer

**Affiliations:** ^1^Nancy Atmospera-Walch School of Nursing, University of Hawaii at Manoa, Honolulu, HI, United States; ^2^College of Nursing, University of South Florida, Tampa, FL, United States; ^3^College of Nursing, University of Tennessee-Knoxville, Knoxville, TN, United States; ^4^College of Medicine Pediatrics, Morsani College of Medicine, University of South Florida, Tampa, FL, United States

**Keywords:** gut microbiome, gut-brain axis, childhood, behavior, gastrointestinal microbiome, microbiota, CBCL scores

## Abstract

**Introduction:**

Very Low Birth Weight (VLBW) infants, born weighing less than 1,500 grams, are at risk for both gut dysbiosis and later neuropsychological developmental deficits. Behavioral effects, while related to neurodevelopment, are often more subtle and difficult to measure. The extent of later neurobehavioral consequences associated with such microbial dysbiosis has yet to be determined. We explored associations between the infants’ gut microbiome and early childhood behavior at 4 years of age and identified the bacterial taxa through a multivariate analysis by linear models.

**Methods:**

Parents completed the Child Behavior Checklist (CBCL) focused on different DSM diagnostic categories: affective, anxiety, pervasive developmental, attention deficit/hyperactivity, and oppositional defiant. All the CBCL scores were corrected for gender, delivery method, gestational age, infant birth weight, occurrence of sepsis, and days on antibiotics prior statistical analyses. Canonical correlation analysis (CCA) was performed to determine the relationship between early life gut microbiome and the adjusted CBCL scores. The association of bacterial Amplicon sequence Variants (ASVs) to the CBCL scores were tested with multivariate analysis by linear models (MaAsLin).

**Results:**

Nineteen children who were previously born with very low birth weight and studied while hospitalized in the Neonatal Intensive Care Unit (NICU) were included in this study. Statistically significant associations were observed between early life gut bacteria such as *Veillonella dispar, Enterococcus, Escherichia coli*, and *Rumincococcus* to later behavior at 4 years. No significant association could be observed with early-life gut microbiome alpha diversity and behavioral measures at 4 years.

**Discussion:**

These preliminary observational data provide insight into the relationships between VLBW gut microbiome dysbiosis and childhood behavior. This study contributes to the literature on gut microbiome analysis by examining various behavioral domains using a standardized tool linked to the Diagnostic and Statistical Manual of Mental Disorders (DSM).

## 1 Introduction

Very Low Birth Weight (VLBW) infants confront unique developmental challenges and a heightened risk of experiencing behavioral issues in their future. Their premature birth means that their organ systems, including the brain, might not have fully matured, leading to potential long-term consequences for neurodevelopment (1, 2). Furthermore, many VLBW infants undergo extended stays in the neonatal intensive care unit (NICU), which can result in sensory deprivation and emotional stress during a critical phase of brain development. Additionally, VLBW infants are more prone to medical complications, such as respiratory distress syndrome, intraventricular hemorrhage, and infections, all of which can have lasting impacts on both their brain, behavior, and growth (3–5). Therefore, it is crucial to identify potential biomarkers or other biological patterns to recognize these challenges early in life.

Numerous studies have illuminated the gut-brain axis, involving bidirectional communication between the gut and the brain, which directly and indirectly impacts the host’s growth, development, and behavioral functions (6–8). Consequently, comprehending the role of gut microbiota in infants could be pivotal for averting the future risk of behavioral issues by enabling the provision of suitable nutrition and early intervention.

Our previous study demonstrated a relationship between the gut microbiota of Very Low Birth Weight (VLBW) and neurodevelopment, assessed using the Battelle Development Inventory-2 Screening Test (BDI-2ST) (2). Behavioral effects, while related to neurodevelopment, are often more subtle and difficult to measure. The use of a parent qualitative scale to describe child behavior is a nuanced approach used in the current study. The Child Behavior Checklist (CBCL) is a standardized tool with six scales related to Diagnostic and Statistical Manual of Mental Disorders (DSM) diagnostic categories. The CBCL has been widely for many years and has shown a high accuracy of diagnostic efficiency (9).

The VLBW infants in this study experienced significant gut microbial dysbiosis during the first 6 weeks of life in the Neonatal Intensive Care Unit (NICU) which was characterized by a dominant abundance of Gammaproteobacteria (10). They are more likely to suffer various brain insults and injuries in their early life. Immaturity, coupled with the intensive care that is necessary, predisposes these infants to gut dysbiosis, a disequilibrium of the gut microbial community (11, 12). The roles of the dysbiotic infant gut microbiome in later childhood neurodevelopment and behavior are understudied. Pathogens present during sensitive developmental periods are associated with later anxiety-like behavior and cognitive impairment (13–16). This may happen because proinflammatory bacterial metabolites from the gut can alter the blood brain barrier or cross into the brain, altering microglia, and contributing to the development of neurological injury (17) which then translates into later neurodevelopmental and behavioral problems. Intestinal dysbiosis often includes reduced microbial alpha diversity and increased intestinal barrier permeability (18). Lower alpha diversity is often correlated to poorer health status (19, 20). The stability, diversity, and developmental succession of the early life gut microbiome may be associated with long-term health consequences (21).

This study explored associations between the VLBW infant’s gut microbiome and scales related to the Diagnostic and Statistical Manual of Mental Disorders (DSM) from the Child Behavior Checklist (CBCL) at 4 years old. We also identified the bacterial Amplicon Sequence Variants (ASVs) related to the DSM-related scores. This study adds to the gut microbiome analysis literature by including analysis related to different behavioral DSM-related scales using a standardized tool.

## 2 Materials and methods

### 2.1 Study design and participants

Upon approval by the university Institutional Review Board (IRB), parents of VLBW infants admitted to the NICU of a large Florida tertiary care hospital were invited to be in the initial cohort (IRB#Pro00003468, R21 NR013094). Parents gave written informed consent to participate in the study and in additional follow up studies. Eighty-three VLBW infants were measured during the first 6 weeks of their NICU admission. Parents who consented were contacted for the follow-up study (IRB#Pro00019955, NIH grant R01NR015446) that explored relationships between the gut microbiome and later health, growth, and development. A total of 25 VLBW infants were followed from birth to 4 years of age. Home visits were done, and multiple types of data were collected. In the current paper, we report on data collected in the NICU, including stool microbiome data, and later behavioral outcomes at 4 years of age. In 19 cases, there were complete data from the 5 and 6 weeks of life for the microbiome analysis, adjustments, and behavioral follow-ups at 4 years of age. Supplementary Figure 1 shows a flow diagram describing clearly the longitudinal follow up design.

### 2.2 Sample processing for measurement of infant and childhood follow up of stool microbiome

Infant stool samples were collected weekly from diapers during the first 6 weeks of life and aliquots were stored at −80 C prior to sequencing. At the 4 year home visit the investigators collected stool samples from the children and their mothers. Stool was collected from the diaper or from the toilet using the ALPCO Easy Sampler^®^ Stool Collection kit. The stool was delivered to the lab and immediately frozen at −80 C until processing for DNA extraction. Microbial genomic DNA was extracted using the PowerSoil DNA Isolation Kit (MoBio) (22). The microbial content was profiled by one contiguous region of 16S rRNA V3-V4 sequencing on an Illumina MiSeq that generated ∼100,000 250 bp paired-end reads per sample. Sequencing quality was assessed, errors corrected, Amplicon Sequence Variants (ASVs) were generated, and their taxonomic annotations were obtained against Silva v138 using the DADA2 pipeline (23). ASVs were used to calculate the alpha diversity, which measures the bacterial diversity as a function of richness and evenness within each sample. For all statistical analyses, only the most abundant ASVs in the dataset were utilized, wherein all ASVs with less than 0.01% abundance in all samples, and ASVs observed in less than 10% of the samples were discarded employing the filter_taxa command implemented in OTU table R package (24).

### 2.3 Behavioral measures

Parents completed the Child Behavior Checklist (CBCL) at home visits. The CBCL is a standardized instrument used to assess behavioral problems in children between 18 and 71 months old (25). It contains 99 items, and each is rated on a three-point Likert scale. This study focused on the six-DSM scales consistent with DSM diagnostic categories: affective, anxiety, pervasive developmental, attention deficit/hyperactivity, and oppositional defiant. The scale is a first level screening, reporting symptoms aligned with diagnostic areas such as autism spectrum disorder (ASD), attention deficit disorder (ADHD), depression, and oppositional defiant disorder (26, 27). Notably, depression symptoms in this age group are manifested mostly as emotional irritability and dysregulation, often differing from manifestations later in development (anhedonia, hopelessness, persistent sadness) (28). The results are age-normed into T-scores with a mean of 50 and standard deviation of 10. Consequently, ranges between 65–69 are considered borderline and scores of 70 or higher are indicative of clinical-range problem (29). A previous publication provides the CBCL descriptive statistics in this sample with means and standard deviations (28).

### 2.4 Statistical analysis

IBM SPSS Statistics version 25 [IBM, (30)] was used to calculate descriptive and frequency statistics for demographic and clinical data. Scores from the CBCL were analyzed using Spearman correlations because of non-normal bivariate distributions.

The microbiome measures were corrected for potential confounding factors by calculating the residual values for each CBCL score after correcting for gender, delivery method, gestational age, infant birth weight, occurrence of sepsis, and days on antibiotics. For all downstream statistical analyses, the residual values of the CBCL scores were utilized. The microbiome data is already available in NCBI database of Genotypes and Phenotypes (dbGaP) with study accession phs001578.v1.p1 (https://www.ncbi.nlm.nih.gov/projects/gap/cgi-bin/study.cgi?study_id=phs001578.v1.p1). The data generated towards achieving the aims of the study are shared through tables and supplementary data described in this study.

### 2.5 Canonical correlation analysis

Canonical correlation analyses (CCA) were performed to identify correlations between infant microbiomes and later behavior. This analysis models correlations between two multivariate sets of data (31). For this purpose, the alpha diversity indices at the infant stage were considered to represent the microbiome, while the residual CBCL values represented the behavioral measures. The R package CCA (version 1.2.1) was utilized for this purpose.

### 2.6 Association of microbiome to later behavior

We examined the associations between early-life microbiome and later behavior (at 4 years) by employing multivariate analysis by linear models (MaAsLin) (32) implemented in the galaxy server (33). The abundant ASVs obtained previously were considered as predictors while the corrected CBCL scores were the outcome. The bacterial counts were converted to relative abundances which were subsequently utilized as input for MaAsLin (34, 35). Apart from ASVs, we further conducted association testing employing MaAsLin for different taxonomic levels including phylum, class, order, family and genus. Associations were considered to be significant if *p* < 0.05.

## 3 Results

### 3.1 Demographics

The 19 children were born early and at very low birth weight (Table 1-sample characteristics and S1-newborn and NICU stay characteristics). They were followed by home visits at 50.3 ± 1.7 months of age. Most were born by Cesarean section (63%), received courses of antibiotics for 15.8 ± 14.8 days, were fed varying amounts of mothers’ own milk and experienced multiple illnesses associated with prematurity. Table 1 provides sample, NICU stay characteristics, and CBCL t-scores at pre-school age.

**TABLE 1:** Population characteristics, neonatal intensive care unit clinical data and DSM-related childhood behavior checklist t-scores.

Family Demographics	Frequency
Maternal education	46.3% high school or less
Marital status	62.5% married
Income	37.9% under 25,000/year
**Ethnicity**	
Caucasian	43.8%
African American	18.8%
Hispanic White	31.3%
Asian	6.3%
**Gender**	
Male	42%
Female	58%
**Delivery method**	
Vaginal	37%
Caesarean section	63%
**NICU events**	
Apgar at 5 min	7.72 ± 1.07
Gestational age (weeks)	27.8 ± 1.7
Birth weight (Gms)	1068.2 ± 215.5
Hemoglobin (Gms/dl)	9.11 ± 1.88
Days on antibiotics	15.8 ± 14.8
Seizures	1 infant
Necrotizing enterocolitis	1 infant
Bronchopulmonary dysplasia	1 infant
Sepsis	1 infant
Intraventricular hemorrhage	1 infant
Retinopathy	2 infants
Days on oxygen	24 ± 26.6
Discharge weight (Gms)	2913.12 ± 1069.1
At preschool age (months)	50.3 ± 1.7
Weight (Kg)	18.3 ± 7.9
Height (cm)	100.25 ± 4.49
Hemoglobin (Gms/dl)	12.09 ± 1.8
Head circumference	
**CBCL scores**	
CBCL1 (depression t score)	56.2 ± 9.2
CBCL2 (anxiety t score)	55.2 ± 8.8
CBCL3 (autism t score)	55.6 ± 7.8
CBCL4 (attention deficit hyperactivity t score)	55.6 ± 8.5
CBCL5 (oppositional/defiant t score)	54.3 ± 8.8

### 3.2 Association between alpha diversity and residual CBCL values

**FIGURE 1:**
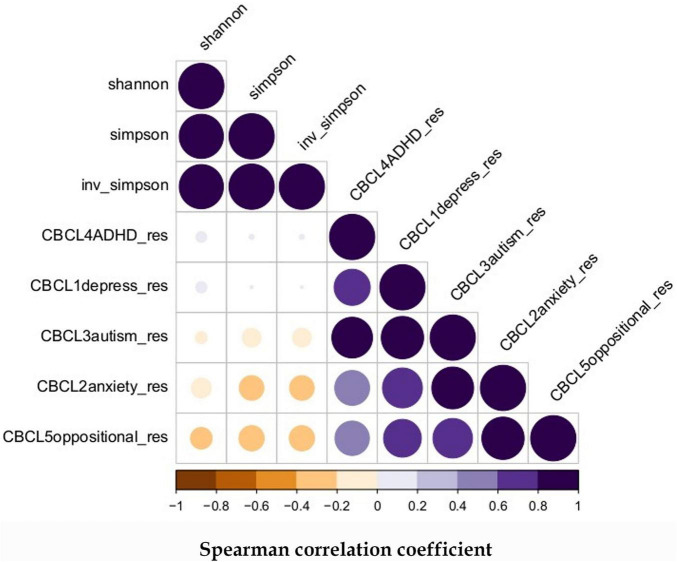
Spearman correlation between microbiome alpha diversity and later childhood behavior: The alpha diversity were derived from ASVs distribution in the samples and all the CBCL scores were adjusted for gender, delivery method, gestational age, infant birth weight, occurrence of sepsis, and days on antibiotics.

Our previous publications (3–5) have summarized the microbiome features obtained in the cohort. For the samples included in this study, we have obtained 103 unique ASVs whose classifications are listed in Supplementary Table 1. The correlations between the alpha diversity and the adjusted CBCL scores are shown in Figure 1. Strong positive correlations were evident among the different CBCL scores but the associations between the alpha diversity measures and CBCL scores were not statistically significant although weak negative correlations were observed between them. The Spearman rank correlation between the adjusted CBCL scores and the alpha diversity measures are listed in Supplementary Table 1, while the corresponding *p*-values are listed in Supplementary Table 2.

### 3.3 Canonical correlation analysis

**FIGURE 2:**
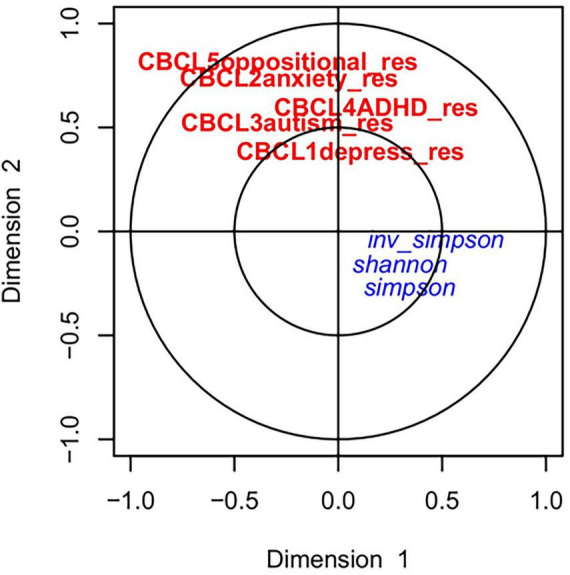
Canonical correlation analyses between alpha diversity at early life and childhood behavior at 4 years: The red and blue color represents the CBCL scores and alpha diversity, respectively. No strong associations between the CBCL scores and alpha diversity were observed.

**FIGURE 3:**
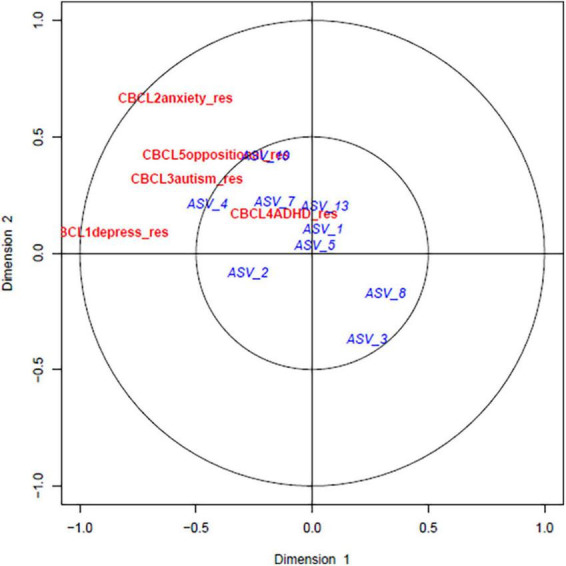
Canonical correlation analyses between predominant bacterial ASVs at early life and childhood behavior at 4 years: The red and blue color represents the CBCL scores and ASVs, respectively. ASV_1 (family *Enterobacteriaceae*), ASV_5 (*Streptococcus*), ASV_7 (*Staphylococcus*) and ASV_13 (*Enterococcus*) was all strongly associated with CBCL_ADHD adjusted values. Similarly, ASV_10 (*Citrobacter*) was related to CBCL_oppositional. ASV_4 (*Enterobacteriaceae*) was associated with both CBCL autism and CBCL ADHD adjusted scores.

The relationship between the gut microbiome parameters in early life was compared with the adjusted CBCL scores at 4 years using canonical correlation analysis (CCA). As shown in Figure 2 the adjusted CBCL measures were highly related to each other but not with the alpha diversity at early life. However, upon investigating the CCA between CBCL scores and prominent ASVs, close relationships were discovered as shown in Figure 3. For example, ASV_1 (family *Enterobacteriaceae*), ASV_5 (*Streptococcus*), ASV_7 (*Staphylococcus*) and ASV_13 (*Enterococcus*) were all highly associated with CBCL_ADHD adjusted values. Similarly, ASV_10 (*Citrobacter*) was related to CBCL_oppositional. ASV_4 (*Enterobacteriaceae*) was associated with both CBCL autism and CBCL ADHD adjusted scores.

### 3.4 Association of early-stage microbiome to behavior at 4 years

Several significant associations were observed between different ASVs and the CBCL scores. In the domain of depression, there was a positive association with *Veillonella dispar* (*p* = 0.0007) and *Escherichia coli* (*p* = 0.02), whereas *Enterococcus* (*p* = 0.03) and *Ruminococcus* (*p* = 0.04) exhibited negative associations. Conversely, in the domain of anxiety, a positive association was observed with *Enterococcus* (*p* = 0.04), while *Veillonella dispar* displayed a negative association (*p* = 0.01). They are summarized in Table 2. The Supplementary Table 3 lists all the associations exported by MaAsLin with the difference CBCL scores. However, for other taxonomic levels including phylum, class, order, family and genus, we could not observe significant associations and the results are listed in Supplementary Table 4.

**TABLE 2:** Association between early-life microbiome and later behavior at 4 years.

Variable	Feature	Bacterial_classification	Coefficient	*P*-value
CBCL1depression	ASV_24	*Veillonella.dispar*	0.024101364	0.000768758
CBCL1depression	ASV_2	*Escherichia coli*	0.086191646	0.024760203
CBCL1depression	ASV_179	*Enterococcus*	-0.005181727	0.038295599
CBCL1depression	ASV_169	*Escherichia coli*	0.045086345	0.038546534
CBCL1depression	ASV_1227	*Ruminococcus*	-0.010014289	0.042389942
CBCL2anxiety	ASV_24	*Veillonella.dispar*	-0.017270338	0.013292532
CBCL2anxiety	ASV_179	*Enterococcus*	0.005296564	0.042749136

Only significant associations are listed.

## 4 Discussion

The composition of the gut microbiome showed significant relationships with DSM-based behavioral scales from the CBCL. In this sample, the alpha diversity was not significantly associated with the adjusted CBCL scores. However, specific Amplicon Sequence Variants analyses were significantly associated with the adjusted CBCL scores, some with positive and some with negative associations.

Enterococcus and *Veillonella dispar* showed significant associations with the CBCL adjusted scales of depression and anxiety. Additionally, there was a significant association of the anxiety CBCL scale with the presence of *Escherichia Coli* and *Ruminococcus*.

Aerobic microbes such as *Enterococcus* and *Escherichia* are the first to colonize the newborn under normal conditions, with the shift occurring to more anaerobic microbes (including *Veillonella*) by 4 months of age (36). However, our sample had high abundance of *Veillonella* within the first weeks in the NICU. Additionally, children born via C-section, which was true for most of the infants, have a higher pathogen abundance of *Klebsiella* and *Enterococcus*, which are associated with a later higher incidence of respiratory infections within the first year of life (37).

The gut microbiome’s relevance to mood disorders is supported by the relationship of microbes with mechanisms affecting mood and behavior. *Enterococcus faecalis* converts naturally occurring levodopa into dopamine via a decarboxylation reaction. Because levodopa is able to cross the blood-brain barrier, but not dopamine, the conversion may lead to reduced central dopamine availability in the brain (38). *Veillonella* abundance has been associated with negative emotions at school age and to cognitive outcomes from birth to adolescence (39). *Veillonella* has also been linked to increased stress levels, as indicated by negative events and emotions reported by parents, emotional problems and low happiness reported by children, and a parasympathetic response to stress. These findings were independent of age, gender, parental education, BMI z-score, fiber, protein, sweet and fat food intake, physical activity, and sleep (40). The links between behavior and ASVs exists, but the direction may differ. Further research is necessary to understand better how gut bacteria and moods are connected. Studies that follow individuals over time would be beneficial in exploring these relationships.

A lower abundance of *Ruminococcus* was related to higher scores in the depression DSM-related scale of the CBCL. Major Depressive Disorder in adults was associated with lower abundance of *Ruminococcus* (41). Contradictory results have been reported for other taxa, but not for *Ruminococcus* (41), indicating that this microbe may be a potential biomarker for depressive disorder.

These children, as infants in the NICU, had dysbiotic gut microbiomes with an overabundance of *Gammaproteobacteria* (10). Dysbiosis could disrupt the normal gut-brain axis in developing infants. Gut microbial products reach the blood and then the brain. These chemicals are capable of modulating neuronal signaling and possibly neurodevelopment. Metabolites of bacterial origin were found in blood samples of children with autism spectrum disorder (ASD), which may cause oxidative stress, mitochondrial dysfunction, and structural changes in the amygdala, cortex, hippocampus, and cerebellum (42).

In conclusion, it is hypothesized that a connection exists between the gut microbiome and behavior. However, most previous studies (43–45) have concentrated on term infants and assessed behavior at 2 years of age or even earlier, which might not entirely align with the conditions at 4 years. Our aim was to investigate gut dysbiosis in VLBW infants and explore the relationships between the early-life gut microbiome and behavior during the preschool years at the age of 4. There were significant relationships between gut microbiome ASVs and DSM-based behavioral scales from the CBCL. Adjusted CBCL scores were significantly associated with ASVs representing *Veillonella dispar, Enterococcus, E. coli*, and *Rumincococcus*. It appears that the gut microbiome dysbiosis of VLBWs may have relationships to later childhood behavior. This study contributes to the gut microbiome literature by adding analyses related to different behavioral domains using a standardized tool linked to the DSM.

These results are preliminary due to the limited sample size. Other factors aside from gender, delivery method, gestational age, infant birth weight, occurrence of sepsis, and days on antibiotics are potentially important in these later childhood relationships. These include human milk, growth, parenting, and development characteristics after discharge.

Dysbiosis in the gut microbiome has shown associations with diverse behavioral disorders, encompassing conditions such as anxiety, depression, and even neurodevelopmental disorders like autism. A comprehensive understanding of the mechanisms through which the infant gut microbiome influences these disorders is of paramount importance for timely intervention and proactive prevention strategies.

While this pilot study offers insights into the association between infancy gut dysbiosis and preschool behavioral functions, a limitation of this manuscript is the small sample size utilized in the study. To validate these findings, further investigation with a larger sample size is necessary. Additionally, there may be other potentially significant factors in these later childhood relationships, such as human milk, growth, parenting, and developmental characteristics after discharge, which were not accounted for in this study.

## Author’s note

SO also worked on this research while affiliated with the University of South Florida, College of Nursing and the University of Tennessee-Knoxville, College of Nursing. AS, JY, and MG also worked on this research while affiliated with the University of South Florida, College of Nursing.

## Data availability statement

The datasets presented in this study can be found in online repositories. The names of the repository/repositories and accession number(s) can be found below: https://www.ncbi.nlm.nih.gov/gap/, phs001578.

## Ethics statement

The studies involving humans were approved by the University of South Florida Institutional Review Board. The studies were conducted in accordance with the local legislation and institutional requirements. Written informed consent for participation in this study was provided by the participants’ legal guardians/next of kin.

## Author contributions

SO: Conceptualization, Data curation, Formal analysis, Investigation, Methodology, Project administration, Resources, Software, Supervision, Validation, Visualization, Writing-original draft, Writing-review and editing. AS: Conceptualization, Data curation, Formal analysis, Investigation, Methodology, Project administration, Resources, Software, Supervision, Validation, Visualization, Writing-original draft, Writing-review and editing. JY: Investigation, Methodology, Resources, Validation, Writing-review and editing. ES-H: Conceptualization, Methodology, Resources, Validation, Writing-review and editing. MG: Conceptualization, Formal analysis, Funding acquisition, Investigation, Methodology, Project administration, Resources, Supervision, Validation, Visualization, Writing-original draft, Writing-review and editing.

## References

[1] Cutland C Lackritz E Mallett-Moore T Bardají A Chandrasekaran R Lahariya C Brighton collaboration low birth weight working group low birth weight: case definition & guidelines for data collection, analysis, and presentation of maternal immunization safety data. *Vaccine.* (2017) 35(48 Pt. A):6492–500. 29150054 10.1016/j.vaccine.2017.01.049 PMC5710991

[2] Sarkar AP Dutra S Youn Yoo J Gordon J Shaffer E McSkimming D Relationships of the very low birth weight infant microbiome with neurodevelopment at 2 and 4 years of age. *Dev Psychobiol.* (2022) 64:e22317. 10.1002/dev.22317 36282736 PMC9608354

[3] Sanders MR Hall SL. Trauma-informed care in the newborn intensive care unit: promoting safety, security and connectedness. *J Perinatol.* (2018) 38:3–10. 28817114 10.1038/jp.2017.124 PMC5776216

[4] Shaw RJ Givrad S Poe C Loi EC Hoge MK Scala M. Neurodevelopmental, mental health, and parenting issues in preterm infants. *Children.* (2023) 10:1565. 37761526 10.3390/children10091565 PMC10528009

[5] Groer M Miller EM Sarkar A Dishaw LJ Dutra SV Youn Yoo J Predicted metabolic pathway distributions in stool bacteria in very-low-birth-weight infants: potential relationships with NICU faltered growth. *Nutrients.* (2020) 12:1345. 32397161 10.3390/nu12051345 PMC7284701

[6] Martin CR Osadchiy V Kalani A Mayer EA. The brain-gut-microbiome axis. *Cell Mol Gastroenterol Hepatol.* (2018) 6:133–48. 30023410 10.1016/j.jcmgh.2018.04.003 PMC6047317

[7] Luna R Savidge T Williams K. The brain-gut-microbiome axis: What role does it play in autism spectrum disorder? *Curr Dev Disord Rep.* (2016) 3:75–81. 27398286 10.1007/s40474-016-0077-7 PMC4933016

[8] Cryan JF O’Riordan KJ Cowan CS Sandhu KV Bastiaanssen TF Boehme M The microbiota-gut-brain axis. *Physiol Rev.* (2019) 99:1877–2013. 31460832 10.1152/physrev.00018.2018

[9] Skarphedinsson G Jarbin H Andersson M Ivarsson T. Diagnostic efficiency and validity of the DSM-oriented child behavior checklist and youth self-report scales in a clinical sample of Swedish youth. *PLoS One.* (2021) 16:e0254953. 10.1371/journal.pone.0254953 34293000 PMC8297893

[10] Yee A Miller E Dishaw L Gordon J Ji M Dutra S Longitudinal microbiome composition and stability correlate with increased weight and length of very-low-birth-weight infants. *mSystems.* (2019) 4:e229–218. 10.1128/mSystems.00229-18 30834328 PMC6392092

[11] Aylward GP. Neurodevelopmental outcomes of infants born prematurely. *J Dev Behav Pediatr.* (2014) 35:394–407. 25007063 10.1097/01.DBP.0000452240.39511.d4

[12] D’Agata AL Wu J Welandawe MK Dutra SV Kane B Groer MW. Effects of early life NICU stress on the developing gut microbiome. *Dev Psychobiol.* (2019) 61:650–60. 30697700 10.1002/dev.21826 PMC6588487

[13] Diaz Heijtz R. Fetal, neonatal, and infant microbiome: Perturbations and subsequent effects on brain development and behavior. *Semin Fetal Neonatal Med.* (2016) 21:410–7. 10.1016/j.siny.2016.04.012 27255860

[14] Goehler L Park S Opitz N Lyte M Gaykema R. Campylobacter jejuni infection increases anxiety-like behavior in the holeboard: Possible anatomical substrates for viscerosensory modulation of exploratory behavior. *Brain Behav Immun.* (2008) 22:354–66. 10.1016/j.bbi.2007.08.009 17920243 PMC2259293

[15] Manco M. Gut Microbiota and Developmental Programming of the Brain: From Evidence in Behavioral Endophenotypes to Novel Perspective in Obesity. *Front Cell Infect Microbiol.* (2012) 2:109. 10.3389/fcimb.2012.00109 22912939 PMC3419354

[16] Sullivan R Wilson D Feldon J Yee B Meyer U Richter-Levin G The international society for developmental psychobiology annual meeting symposium: Impact of early life experiences on brain and behavioral development. *Dev Psychobiol.* (2006) 48:583–602. 10.1002/dev.20170 17016842 PMC1952656

[17] Volpe J. Encephalopathy of prematurity includes neuronal abnormalities. *Pediatrics.* (2005) 116:221–5. 10.1542/peds.2005-0191 15995055

[18] Harbison JE Roth-Schulze AJ Giles LC Tran CD Ngui KM Penno MA Gut microbiome dysbiosis and increased intestinal permeability in children with islet autoimmunity and type 1 diabetes: A prospective cohort study. *Pediatr Diab.* (2019) 20:574–83. 10.1111/pedi.12865 31081243

[19] Moran-Ramos S Lopez-Contreras BE Villarruel-Vazquez R Ocampo-Medina E Macias-Kauffer L Martinez-Medina JN Environmental and intrinsic factors shaping gut microbiota composition and diversity and its relation to metabolic health in children and early adolescents: a population-based study. *Gut Microbes.* (2020) 11:900–17. 31973685 10.1080/19490976.2020.1712985 PMC7524342

[20] Prehn-Kristensen A Zimmermann A Tittmann L Lieb W Schreiber S Baving L Reduced microbiome alpha diversity in young patients with ADHD. *PLoS One.* (2018) 13:e0200728. 10.1371/journal.pone.0200728 30001426 PMC6042771

[21] Sarkar A Yoo JY Valeria Ozorio Dutra S Morgan KH Groer M. The association between early-life gut microbiota and long-term health and diseases. *J Clin Med.* (2021) 10:459. 33504109 10.3390/jcm10030459 PMC7865818

[22] QIAGEN. *MO BIO’s Powersoil DNA Isolation Kit Handbook: QIAGEN.* (2013). Available online at: https://www.qiagen.com/us/resources/resourcedetail?id=5c00f8e4-c9f5-4544-94fa-653a5b2a6373&lang=en (accessed March 2017).

[23] Callahan B McMurdie P Rosen M Han A Johnson A Holmes S. DADA2: High-resolution sample inference from Illumina amplicon data. *Nat Methods.* (2016) 13:581–3. 10.1038/nmeth.3869 27214047 PMC4927377

[24] Linz A Crary B Shade A Owens S Gilbert J Knight R Bacterial community composition and dynamics spanning five years in freshwater bog lakes. *mSphere.* (2017) 2:169–117. 10.1128/mSphere.00169-17 28680968 PMC5489657

[25] Achenbach T. *Manual for the Teacher’s Report Form and 1991 profile.* Burlington: University of Vermont Department of Psychiatry (1991).

[26] Arias A Rea M Adler E Haendel A Van Hecke A. Utilizing the child behavior checklist (CBCL) as an Autism spectrum disorder preliminary screener and outcome measure for the peers^®^ intervention for autistic adolescents. *J Autism Dev Disord.* (2021) 52:2061–74. 10.1007/s10803-021-05103-8 34052960 PMC9926906

[27] So P Greaves-Lord K van der Ende J Verhulst F Rescorla L de Nijs P. Using the child behavior checklist and the teacher’s report form for identification of children with autism spectrum disorders. *Autism.* (2013) 17: 595–607. 22914776 10.1177/1362361312448855

[28] Dutra SVO Gordon J Shaffer E Miller E Harville C Yoo JY An exploratory principal factor analysis of very low birth weight clinical data and development-behavior outcomes at 4 years of age. *Pediatr Nurs*. (2022) 48:21–33.

[29] Achenbach TM Edelbrock C. Child behavior checklist. *Burlington (Vt)*. (1991) 7:371–92.

[30] IBM. *IBM Support: Downloading IBM SPSS Statistics 25*. (2023). Available online at: https://www.ibm.com/support/pages/downloading-ibm-spss-statistics-25 (accessed July 2023).

[31] Thompson B. *Canonical Correlation Analysis. Reading and Understanding MORE Multivariate Statistics*. Washington, DC: American Psychological Association (2000). p. 285–316.

[32] Mallick H Rahnavard A McIver L Ma S Zhang Y Nguyen L Multivariable association discovery in population-scale meta-omics studies. *PLoS Comput Biol.* (2021) 17:e1009442. 10.1371/journal.pcbi.1009442 34784344 PMC8714082

[33] Huttenhower Lab. *Galaxy/Hutlab: Department of Biostatistics. Harvard T.H. chan School of Public Health.* Boston, MA: Huttenhower Lab (2023).

[34] Collins KH Schwartz DJ Lenz KL Harris CA Guilak F. Taxonomic changes in the gut microbiota are associated with cartilage damage independent of adiposity, high fat diet, and joint injury. *Sci Rep*. (2021) 11:14560. 34267289 10.1038/s41598-021-94125-4 PMC8282619

[35] Hoskinson C Zheng K Gabel J Kump A German R Podicheti R Composition and functional potential of the human mammary microbiota prior to and following breast tumor diagnosis. *Msystems*. (2022) 7: e1489–1421. 10.1128/msystems.01489-21 PMC9239270 35642922

[36] Bäckhed F Roswall J Peng Y Feng Q Jia H Kovatcheva-Datchary P Dynamics and stabilization of the human gut microbiome during the first year of life. *Cell Host Microbe.* (2015) 17:690–703. 10.1016/j.chom.2015.04.004 25974306

[37] Reyman M van Houten M van Baarle D Bosch A Man W Chu M Impact of delivery mode-associated gut microbiota dynamics on health in the first year of life. *Nat Commun.* (2019) 10:4997. 10.1038/s41467-019-13014-7 PMC6825150 31676793

[38] Maini Rekdal V Bess E Bisanz J Turnbaugh P Balskus E. Discovery and inhibition of an interspecies gut bacterial pathway for Levodopa metabolism. *Science.* (2019) 364:1055. 10.1126/science.aau6323 31196984 PMC7745125

[39] McMath A Aguilar-Lopez M Cannavale C Khan N Donovan SM. A systematic review on the impact of gastrointestinal microbiota composition and function on cognition in healthy infants and children. *Front Neurosci.* (2023) 17:1171970. 10.3389/fnins.2023.1171970 37389363 PMC10306408

[40] Michels N Van de Wiele T De Henauw S. Chronic psychosocial stress and gut health in children: associations with calprotectin and fecal short-chain fatty acids. *Psychosom Med.* (2017) 79:927–35. 10.1097/PSY.0000000000000413 27787408

[41] Maes M Vasupanrajit A Jirakran K Klomkliew P Chanchaem P Tunvirachaisakul C Exploration of the gut microbiome in thai patients with major depressive disorder shows a specific bacterial profile with depletion of the ruminococcus genus as a putative biomarker. *Cells.* (2023) 12:1240. 10.3390/cells12091240 37174640 PMC10177051

[42] Srikantha P Mohajeri M. The possible role of the microbiota-gut-brain-axis in autism spectrum disorder. *Int J Mol Sci.* (2019) 20:2115. 10.3390/ijms20092115 PMC6539237 31035684

[43] Carlson AL Xia K Azcarate-Peril MA Rosin SP Fine JP Mu W Infant gut microbiome composition is associated with non-social fear behavior in a pilot study. *Nat Commun*. (2021) 12:3294. 34078892 10.1038/s41467-021-23281-y PMC8172562

[44] Loughman A Ponsonby A-L O’Hely M Symeonides C Collier F Tang MLK Gut microbiota composition during infancy and subsequent behavioural outcomes. *EBioMedicine*. (2020) 52:102640. 32062351 10.1016/j.ebiom.2020.102640 PMC7016366

[45] Laue HE Korrick SA Baker ER Karagas MR Madan JC. Prospective associations of the infant gut microbiome and microbial function with social behaviors related to autism at age 3 years. *Sci Rep*. (2020) 10:15515. 32968156 10.1038/s41598-020-72386-9 PMC7511970

