# Supplementary material for: Premature Infant Gut Microbiome relationships with childhood behavioral scales: preliminary insights
Source: Front Nutr. 2024 Feb 14;10:1294549. doi: 10.3389/fnut.2023.1294549 (PMC10899318; doi:10.3389/fnut.2023.1294549)
Supplement: Supplementary file 1 [file Table_1.docx]

**Supplementary table 1: Spearman correlation coefficients for the relationship between adjusted CBCL scores and the measures of alpha diversity.**

|  | CBCL1depress | CBCL2anxiety | CBCL3autism | CBCL4ADHD | CBCL5oppositional | shannon | simpson | inv_simpson |
| --- | --- | --- | --- | --- | --- | --- | --- | --- |
| CBCL1depress | 1.00 | 0.78 | 0.88 | 0.64 | 0.70 | 0.06 | 0.01 | 0.01 |
| CBCL2anxiety | 0.78 | 1.00 | 0.85 | 0.59 | 0.88 | -0.20 | -0.30 | -0.30 |
| CBCL3autism | 0.88 | 0.85 | 1.00 | 0.81 | 0.76 | -0.07 | -0.18 | -0.18 |
| CBCL4ADHD | 0.64 | 0.59 | 0.81 | 1.00 | 0.58 | 0.06 | 0.01 | 0.01 |
| CBCL5oppositional | 0.70 | 0.88 | 0.76 | 0.58 | 1.00 | -0.23 | -0.32 | -0.32 |
| shannon | 0.06 | -0.20 | -0.07 | 0.06 | -0.23 | 1.00 | 0.95 | 0.95 |
| simpson | 0.01 | -0.30 | -0.18 | 0.01 | -0.32 | 0.95 | 1.00 | 1.00 |
| inv_simpson | 0.01 | -0.30 | -0.18 | 0.01 | -0.32 | 0.95 | 1.00 | 1.00 |
