# Supplementary material for: Premature Infant Gut Microbiome relationships with childhood behavioral scales: preliminary insights
Source: Front Nutr. 2024 Feb 14;10:1294549. doi: 10.3389/fnut.2023.1294549 (PMC10899318; doi:10.3389/fnut.2023.1294549)
Supplement: Supplementary file 2 [file Table_2.docx]

**Supplementary table 2: p-values for Spearman correlation coefficients for the relationship between adjusted CBCL scores and the measures of alpha diversity.**

|  | CBCL1depress | CBCL2anxiety | CBCL3autism | CBCL4ADHD | CBCL5oppositional | shannon | simpson | inv_simpson |
| --- | --- | --- | --- | --- | --- | --- | --- | --- |
| CBCL1depress | NA | 9.0E-05 | 9.2E-07 | 0.003 | 0.001 | 0.797 | 0.983 | 0.983 |
| CBCL2anxiety | 9.0E-05 | NA | 3.2E-06 | 0.007 | 0.000 | 0.412 | 0.212 | 0.212 |
| CBCL3autism | 9.2E-07 | 3.2E-06 | NA | 0.000 | 0.000 | 0.764 | 0.464 | 0.464 |
| CBCL4ADHD | 3.4E-03 | 7.2E-03 | 2.8E-05 | NA | 0.009 | 0.814 | 0.955 | 0.955 |
| CBCL5oppositional | 9.2E-04 | 9.2E-07 | 1.4E-04 | 0.009 | NA | 0.340 | 0.188 | 0.188 |
| shannon | 8.0E-01 | 4.1E-01 | 7.6E-01 | 0.814 | 0.340 | NA | 0.000 | 0.000 |
| simpson | 9.8E-01 | 2.1E-01 | 4.6E-01 | 0.955 | 0.188 | 0.000 | NA | 0.000 |
| inv_simpson | 9.8E-01 | 2.1E-01 | 4.6E-01 | 0.955 | 0.188 | 0.000 | 0.000 | NA |
