# Supplementary figures and images for: Premature Infant Gut Microbiome relationships with childhood behavioral scales: preliminary insights
Source: Front Nutr. 2024 Feb 14;10:1294549. doi: 10.3389/fnut.2023.1294549 (PMC10899318; doi:10.3389/fnut.2023.1294549)

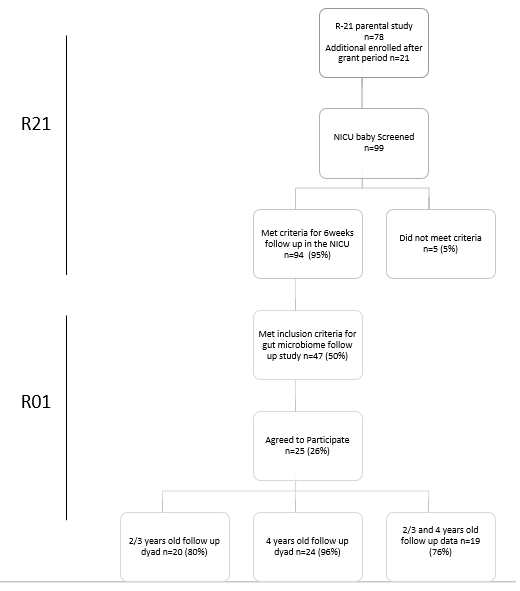

Supplement: Supplementary file 5 [file Image_1.tif]
